# A Cost-Effective Method to Assemble Biomimetic 3D Cell Culture Platforms

**DOI:** 10.1371/journal.pone.0167116

**Published:** 2016-12-09

**Authors:** Sabreen Khalil, Nagwa El-Badri, Mohamed El-Mokhtaar, Saif Al-Mofty, Mohamed Farghaly, Radwa Ayman, Dina Habib, Noha Mousa

**Affiliations:** 1 Center of Excellence for Stem Cells and Regenerative Medicine, Zewail City of Science and Technology, Giza, Egypt; 2 University of Science and Technology, Zewail City of Science and Technology, Giza, Egypt; 3 Department of Microbiology and Immunology, Faculty of Medicine, Assiut University, Assiut, Egypt; 4 Department of Obstetrics and Gynecology, Faculty of Medicine, Assiut University, Assiut, Egypt; Indian Institute of Toxicology Research, INDIA

## Abstract

Developing effective stem cell based therapies requires the design of complex *in vitro* culture systems for more accurate representation of the stem cell niche. Attempts to improve conventional cell culture platforms include the use of biomaterial coated culture plates, sphere culture, microfluidic systems and bioreactors. Most of these platforms are not cost-effective, require industrial technical expertise to fabricate, and remain too simplistic compared to the physiological cell niche. The human amniotic membrane (hAM) has been used successfully in clinical grafting applications due to its unique biological composition and regenerative properties. In this study, we present a combinatorial platform that integrates the hAM with biomolecular, topographic and mechanical cues in one versatile model.

**Methods:**

We utilized the hAM to provide the biological and the three dimensional (3D) topographic components of the prototype. The 3D nano-roughness of the hAM was characterized using surface electron microscopy and surface image analysis (ImageJ and SurfaceJ). We developed additional macro-scale and micro-scale versions of the platform which provided additional shear stress factors to simulate the fluid dynamics of the *in vivo* extracellular fluids.

**Results:**

Three models of varying complexities of the prototype were assembled. A well-defined 3D surface modulation of the hAM in comparable to commercial 3D biomaterial culture substrates was achieved without complex fabrication and with significantly lower cost. Performance of the prototype was demonstrated through culture of primary human umbilical cord mononuclear blood cells (MNCs), human bone marrow mesenchymal stem cell line (hBMSC), and human breast cancer tissue.

**Conclusion:**

This study presents methods of assembling an integrated, flexible and low cost biomimetic cell culture platform for diverse cell culture applications.

## Introduction

Significant number of diseases affecting human health are awaiting successful cell based therapies. A major focus of current cell research is to create effective *in vitro* culture systems to expand or differentiate stem or progenitor cells [1]. Given that stem cell studies have been mostly conducted in flat rigid platforms and static culture media, the outcome of these studies has often failed to show relevance when stem cells were transplanted *in vivo* for therapeutic applications. For example, generating a clinically useful number of undifferentiated cells remains to be a challenge [2]. Likewise, homing and engraftment of stem cells into the target organ and commitment to the desired function pose added difficulties [3]. Such challenges have driven research efforts to mimic the stem cell niche which presents an ecosystem with intricate biological, biophysical, and architectural factors that collectively define the native environment of the cell [4, 5]. The topographic and mechanical niche cues are particularly necessary for maintaining the three dimensional (3D) alignment and spatial orientation of cells. They also enable an effective cell-cell interaction, a key driver of the stem cell fate [6–8]. These factors may also determine critical cell behaviors such as programmed cell death or malignant alteration into a cancer initiating cell [5].

Current biomimetic platforms mostly address a single factor of the cell microenvironment. Furthermore, most biomaterials used for cell culture are fabricated from either synthetic polymers or a single natural compound derived from matrix proteins or adhesion molecules such as collagen, laminin, fibronectin or matrigel. 3D nanofiber networks or micro-patterned arrays of one or a few of the extra cellular matrix (ECM) components have been also used [1, 9]. These approaches remain overly simple as they cannot reproduce the complexity of the niche, and it would be practically and economically impossible to fabricate all native biomolecules into one culture system. Additionally, a considerable technical effort and expertise are involved in immobilizing growth factors on biomaterial surfaces to enhance their cell-to-matrix interactions. As a result, regular polystyrene culture plates continue to be the most used in biological culture systems.

Novel usage of natural substrates, such as the human amniotic membrane (hAM) thus represents an attractive and convenient approach to enrich the biomolecular component of the niche. The hAM has been long used in clinical ocular applications, being accessible as a by-product of delivery that is often disposed in maternity hospitals [10, 11]. The structure of the hAM has been widely investigated [12]. Following its separation from the chorion, it is composed of a superficial single epithelial layer and basement membrane rich in collagen (especially types III, IV and V), laminin, fibronectin, and nidogen. The basement membrane is attached to a compact connective tissue layer, rich in collagen and fibronectin, that rests on a cellular layer of fibroblasts and an outer looser spongy layer. The latter contains collagen (especially type I) and various proteoglycans such as perlecan, keratan, heparan and chondroitin sulphates [8, 9]. Different kinds of growth factors such as keratinocyte growth factor (KGF), basal fibroblast growth factor (b-FGF), hepatocyte growth factor (HGF), transforming growth factor beta (TGF-ß) and many others are produced in the hAM [13, 14]. The antimicrobial and anti-inflammatory properties of the hAM were attributed to its content of matrix metalloproteinases (MMPs) inhibitors, elafin, β -defensin and leukocyte proteinase inhibitor (SLPPI)[15].The suppression of pro-inflammatory cytokines such as IL-1 α and IL-1β by the hAM stroma was also evident[16]. Indeed, during pregnancy, the potent anti-microbial effect of the hAM balances its immunosuppressive properties [17, 18], and provides a perfect equilibrium for the mother by enabling her to tolerate the development of the foreign fetal tissue without undermining her systemic defense against infection. Such outstanding biological profile suggests a potential unique value of the hAM as a biomaterial component of an effective culture system.

In the present study, we developed methods to integrate the hAM into a multifaceted biomimetic cell culture platform that addresses additional key niche factors.

## Materials and Methods

### Preparation of the Human Amniotic Membrane (hAM)

The hAM specimens were obtained from healthy women after delivery of the fetus and clamp-cutting of the umbilical cord. The umbilical cord blood and amniotic membrane samples were obtained at Assiut University Women’s Health Hospital and Sheikh Zayed Hospital between May 2015 and May 2016. The research study protocol was approved by the institutional ethics committees for collecting the routinely disposed amniotic membrane and umbilical cord blood for the study purpose. Accordingly, we obtained samples from 5 women, 2 of them by informed verbal consents and 3 by written consents.

The free side of the amnion was excised, inserted into sterile saline solution, and transferred immediately to the lab. All experiments were run using fresh non-cryopreserved hAM. The amnion layer of the hAM was separated manually or by blunt dissection from the chorion layer. For consistency, it was important to distinguish the glistening epithelial surface of the amniotic membrane from the outer connective tissue interface. The hAM was washed several times by sterile phosphate buffer saline (PBS-1X, w/o Ca++, Mg++, Life technologies, CA, USA) containing 0.1% antibiotic antimycotic (Life technologies, CA, USA). The specimen was divided into small segments of approximately 10 cm diameter each. Each segment was stretched on a homemade cell crown composed of a de-capped container and a gauze membrane to allow homogenous distribution of the reagents over the epithelial surface of the membrane (S1 Fig).

### The hAM Surface Modification

In order to efficiently remove the epithelial cell layer of the amnion and produce a 3D culture surface, we first evaluated previously reported decellularization methods. We compared the use of trypsin-EDTA with mechanical scraping to the use of sodium hydroxide (NaOH), as reported by Saghizadeh et al.[19]. In the trypsin treatment method, trypsin (0.25%, Life technologies; CA, USA) was used to treat the membrane for 90 minutes following a modification of a published protocol [20].In the NaOH treatment method, 2 grams were dissolved in 50 ml of distilled water (working concentration of 40 mg/ml), and applied gently for 30 seconds to 1 minute with the aid of a cotton tip. NaOH was thoroughly washed off for 5 to 10 minutes using sterile PBS. Methylene blue (0.05% solution, Bio-Tek, Egypt), or trypan blue dyes (Trypan Blue 0.4% solution in 0.85% NaCl, Lonza, Basel, Switzerland) were applied to verify the adequacy of cell removal. The stained specimens were examined by bright field microscopy. Non-stained membranes were additionally examined using an inverted microscope (Olympus IX35, Tokyo, Japan).

### Surface Electron Microscope Examination

In order to study the ultra-topography of the membrane surface of both intact and decellularized hAM, SEM examination was performed. Both NaOH-treated membrane and intact membranes were preserved in glutaraldehyde for optimal preservation of tissue ultra-architectural details. The specimens of both groups were molded on a specimen stub and sputter coated with gold in a JEOL–JFC1100E ion sputtering device, and examined by a Surface Electron Microscope (SEM) (JEOL JSM-5400, Tokyo, Japan). Images were obtained at increasing magnifications (x500, x1000, x2000, and x5000).

**Composing the Biomimetic Niche**: Three prototypes were developed to demonstrate the feasibility of composing different models suitable for various cell culture applications.

**Prototype 1:** hAM was decellularized as described above and used to line commercial plastic petri dishes and well culture plates (6, 12, 24 and 96 wells; Greiner Bio-One North America Inc, USA). Coating of the dishes and wells was achieved by direct application of the membrane, so that the epithelial surface faces upwards while the connective tissue side adheres to the plate surface. A 25-gauge needle attached to a 3 cm syringe was used to achieve good attachment by gentle suction of air bubbles beneath the membrane to create a negative pressure.

**Prototype 2:** This prototype was developed to provide an enhanced biomimetic niche. The platform was fabricated using poly dimethyl siloxane (PDMS) (SYLGARD® 184 silicon elastomer kit, Dowcorning, MI, USA). PDMS was prepared by mixing a pre-polymer base and a curing agent (ratio 10:1 w/w). A spherical PDMS container was made with an average thickness of 3–5 mm. A regular urinary latex Foley’s catheter balloon (Generic, China) was inflated while inside an open spherical container made of soft plastic, of 4 cm internal diameter. The two ends of the Foley’s catheter were fixed vertically on the upper and lower borders of a rectangular frame to allow stability of the balloon and the even distribution of PDMS until it is cured around the balloon. Liquid PDMS was then injected slowly and evenly all around the inflated Foley’s balloon filling a thin potential space of approximately 3 mm in thickness. An internal PDMS knob was shaped during the process. After curing the PDMS, inlet and outlet channels were inserted through the container wall to introduce continuous fluid flow using a syringe pump (NE-4000, New Era Pump Systems, Inc., NY, USA). These channels were assembled using various size cannulae and pediatric naso-gastric (Ryle) tubes. The hAM was then decellularized as outlined above and suctioned using a syringe as described in the previous model to adhere firmly to the internal surface of PDMS.

**Prototype 3:** A microfluidic chip was used in this model. The microchip was originally fabricated for the purpose of tissue dissociation in a previous work by our group [21]. The hAM was decellularized as outlined above and small segments (~ 5x10 mm) were used. A micro plug (~2 mm) was excised from cured PDMS and the hAM segment was adhered on its convex surface. The hAM-covered plug was inserted top-down into a microchamber (3 mm diameter) that is connected, through 200 μm straight microfluidic channels, to an inlet and an outlet on the chip.

### Umbilical Cord Blood Derived Mononuclear Cells Culture

Umbilical cord Mononuclear Cells (MNCs) were isolated following a modification of a previously described method [22]. Briefly, human fetal umbilical cord blood (volumes ranged between 20 ml to 50 ml) was collected from the umbilical cord, immediately after clamping, into heparin-containing sterile tubes using aseptic technique. Samples were diluted at a ratio of 1:1 using PBS. The diluted blood was applied to density gradient (Ficoll Histopaque; Sigma-Alderish, USA). Samples were centrifuged at 400g for 30 minutes. The buffy coat containing the MNCs was collected, washed twice by PBS, and centrifuged at 250 g for additional 10 minutes to eliminate platelets. Finally, cells were suspended in 5 ml of DMEM containing 10% FBS (Lonza, Basel, Switzerland) and counted using a hemocytometer. Sterile 12-well plates were coated with a decellularized hAM as described above in prototypes 1 and 2. Experiments were done in triplicates. 0.5 x10^6^ MNCs were plated in each well and the volume was completed to 2 ml using DMEM high glucose containing 10% FBS, and supplemented with 2 mM L-glutamine, 100 U/ml penicillin and 0.1 mg/ml streptomycin. A control group included MNCs cultured in similar wells under the same conditions without hAM. Plates were incubated in a humidified incubator at 37°C and 5% CO2. Cells were observed regularly using the inverted microscope and serial images were taken every 1–2 days. At final evaluation, cultured cells were washed off the membrane and centrifuged for 10 min at 800 g and 10 μL of the cell pellet was counted using a hemocytometer.

### Proliferation and Differentiation of Human Bone Marrow Mesenchymal Stem Cells

Human bone marrow mesenchymal stem cells (hBMSC) (ATCC ® PCS-500-012, ATCC Inc., USA) were cultured in prototype 1 (prepared using decellularized hAM as described above using a 96-well plate). After initial culture in regular plates for 5 days, 2000 cells/well were seeded, in 5 replicate samples, in each of the hAM coated and non-coated wells. DMEM low glucose supplemented with 10% FBS and penicillin/streptomycin were used to compose a complete culture medium (CCM) for this culture. The Alamar Blue® cell proliferation and viability assay (10X, Invitrogen, USA) was performed to compare cell proliferation and metabolic activity in both groups. After 24 hours of incubation at standard culture conditions, the Almar Blue reagent (20 μL) was added to each well. The plate was covered by aluminum foil and incubated for further 4 hours at 37°C 5% CO2. We have used a plate reader (FLUstar Omega, Germany) to measure the absorbance of Alamar Blue® at 570 nm, using 600 nm as a reference wavelength. Proliferation of cells was calculated based on the relative percent (%) reduction of the Alamar Blue reagent in the hAM based culture compared to the average % reduction in the control wells. The equation used is available as supplementary information (S2 File).

#### Stem Cell Differentiation Assays

hBMSC were seeded at a density of 30,000 cells per well in the hAM coated prototype 1, and similarly in control regular wells of a 6-well plate. After initial cell adherence overnight, 1 ml of osteogenic or adipogenic differentiation media was added to each well. The osteogenic differentiation medium consisted of CCM prepared from DMEM low glucose, 10% FBS, penicillin/streptomycin (1000U/ml and 250mg/ml), and 1x L-Glutamine which was supplemented with B-glycerol phosphate (10 μM, Serva, Netherlands), L-ascorbic acid (50ug/ml, Sigma, USA), and dexamethasone (100 nM, Lonza, USA). The adipogenic differentiation medium consisted of CCM supplemented with dexamethasone (1 μM), bovine insulin (5ug/ml, Sigma, USA), indomethacin (50 μM Alfa Aesar, USA), and 3-Isobutyl-methylxanthine (IBMX) (0.5 μM, Sigma, USA). The media were carefully topped up every 2–3 days. Additional group of wells (hAM coated and non-coated) were supplemented with only CCM as a control. After 21 days of incubation at standard conditions, differentiation media were aspirated and wells were washed three times with PBS. Cells were incubated with 70% ethanol for 1 hour at room temperature, then washed twice with MilliQ water (Merk Millipore, USA). Water was removed and 3ml of Alazirin Red (4.1–4.3 pH 4.13, Alpha Chemika, India) was used to stain the calcium deposits as an evidence of osteogenic differentiation.

For adipogenic differentiation, Oil Red O (150 mg Oil Red O in 50 ml 99% isopropanol, Alpha Chemika, India) was used to stain the fat droplets for confirmation of adipogenic differentiation. Similarly, after 21 days, differentiation media were aspirated and wells were washed three times with PBS. Cells were incubated for 1 hour at room temperature with 10% formaldhyde (ThermoFisher, USA), then washed twice with water. Water was aspirated and isopropanol (Sigma, Germany) was added for 5 minutes. Isopropanol was then aspirated and 24 ml Oil Red O was added to 16 ml of distilled water (3:2 v/v dilution ratio). The stained cells were incubated for 15 minutes at room temperature, washed with water till it is clear, then all water was discarded. All plates were examined using an inverted microscope (Leica DMi8, Germany).

### Breast Cancer Explant Culture

Breast cancer tissue was obtained from one patient after mastectomy, following ethics approval and patient’s informed written consent. Ethics approval was obtained from South Egypt Oncology Institute at Assiut University. A small piece of the tumor tissue was cut into 1–2 mm fragments and cultured on an intact hAM in prototype 1. Similar fragments of the same tumor were cultured on the same day in serum coated 6-well plates as previously described [23], without the use of the hAM. Humec ready medium (GIBCO® HuMEC, Invitrogen, US), a specific medium for optimizing the growth of breast epithelial cells, was used (1–2 ml/well). Antibiotic-antimycotic and minimal amount of FBS (0.5%) were added. Both groups were maintained under similar culture conditions and examined regularly.

### Surface Topography Image Analysis

An image analysis method was applied to demonstrate the ultra-topography of the amniotic membrane after decellularization. ImageJ of the National Institute of Health (http://imagej.nih.gov/ij/) was used to analyze SEM images of the highest magnification obtained in this study (x 5000). Images were analyzed in the grey scale 8-bit mode. The plug-in Interactive 3D Surface Plot was applied with optimization of display options using the exact parameters in all compared images. Additionally, we installed SurfaceJ plugin to ImageJ. SurfaceJ was used to quantitatively characterize the topographic nano-roughness of the hAM and randomly selected synthetic scaffolds used as 3D substrates for cell culture. Images for the latter group were obtained from publications of the manufacturer or the research group fabricating them. All images were then converted to (32-bit grey scale) and the command *Plugins/Analyze/SurfCharJ q1* was used. To standardize the characterization method for all compared images, we used 130 pixels as a sampling length and surface leveling was applied for all analyses.

### Statistical Analysis

Excel (Microsoft, USA) was used to record data, calculate mean, SD, CV%, and create graphs of proliferation studies. Statistical Package for the Social Science (SPSS) (IBM, USA) was used for statistical analysis. Mann-Whitney U test was applied to compare the independent variables of cell proliferation studies based on either cell count or % reduction of the OD values. P value was set significant at a p <0.05.

## Results

### Decellularization of the hAM

**Fig 1:**
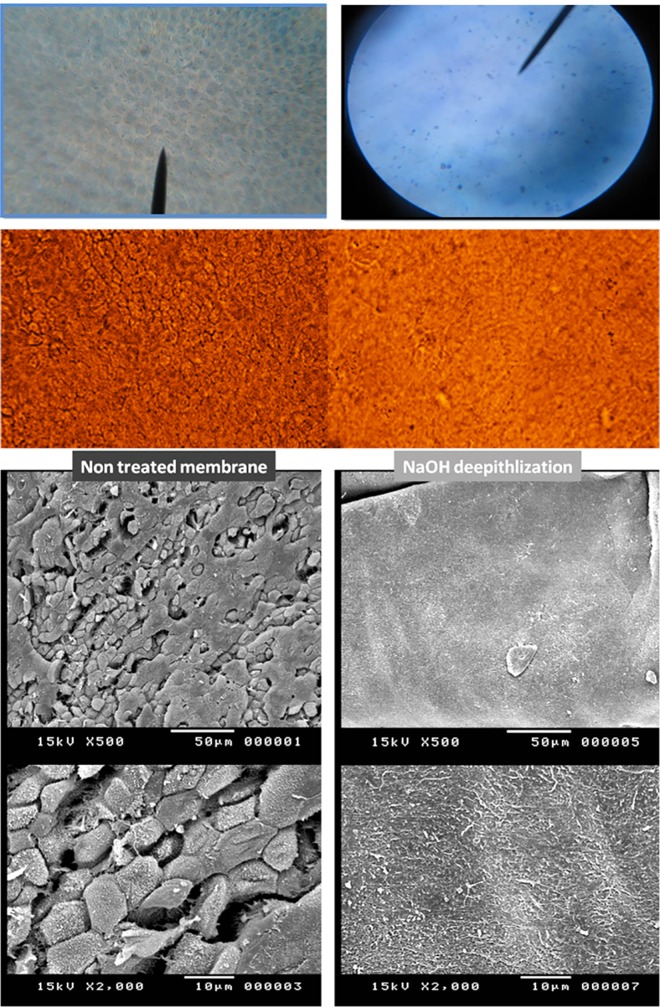
Human Amniotic Membrane Surface Treatment. This figure shows the fetal side of the hAM in its intact (left column) and decellularized (right column) states. Complete decellularization was achieved in repeat experiments using the brief alkaline method as demonstrated by various imaging modalities and magnifications (Top lane shows bright field microscopy images where hAM stained with methylene blue, second lane shows inverted microscopy images followed by SEM images at x500 and x2000).

Complete decellularization of the membrane was consistently reproduced and has been confirmed using various imaging techniques including bright field microscopy, inverted microscopy, and SEM (Fig 1). Decellularization of the hAM following the NaOH treatment protocol was faster (required only 1 minute) and appeared to be more efficient than trypsin treatment, alone or in combination with sequential NaOH treatment. The latter resulted in prolonged exposure to trypsin for 30 minutes after which only partial decellularization was achieved (S2 Fig).

### The Biomimetic Platform

**Fig 2:**
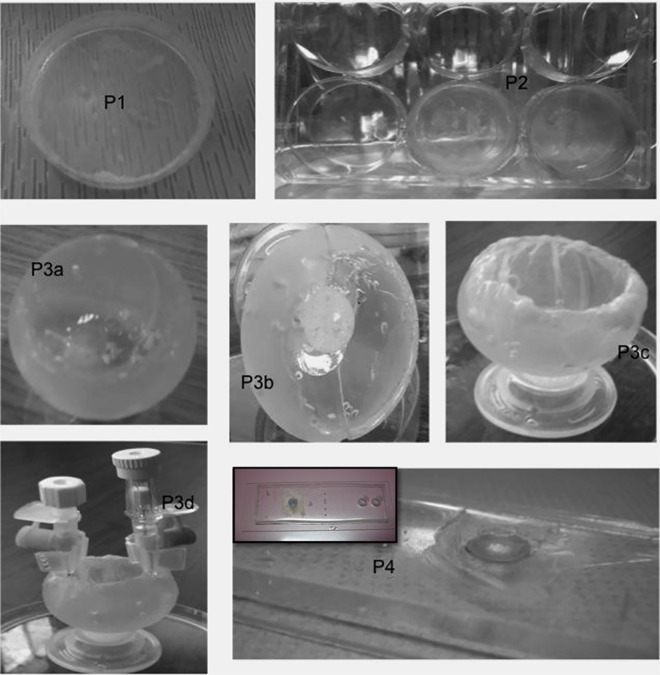
Prototypes of the Biomimetic Platform. Various examples of the prototypes are shown. P1 and P2 are platforms in which regular Petri dishes or well plates lined with hAM were used. P3 shows successive steps of developing a PDMS-hAM platform with an additional fluid dynamic factor (a-d). P4 is a microchip chamber connected with microfluidic channels (inlet and outlet channels), the hAM layer was applied top-down to allow the free flow of media across the chamber.

Different prototypes were constructed and tested for basic functionality as shown in (Fig 2). In prototype 1; we have used the treated hAM as a coating for traditional cell culture plates. Accordingly, these 2D regular plates and wells were upgraded into a 3D platform following a few simple steps (Fig 2- P1, P2). In prototype 2, we produced a 3D custom made culture platform that can be shaped into any design and size utilizing the moldability of both the PDMS and the hAM. We enabled a dynamic flow of the culture media into prototype 2 using external tubing attached to a syringe pump. We have successfully maintained the flow for 7 days (for 5 continuous hours per day, at a flow rate of 1 ml/hour) (Fig 2- P3: a-d). In prototype 3, which is based on a hybrid microfluidics-hAM model, the microchamber was turned into a culture reservoir equipped with a 3D surface in its upper “hAM coated” section (Fig 2- P4). A dynamic culture media could freely flow through the built-in microchannels across the lower section of the chamber. For demonstration, we introduced a continuous flow of saline followed by trypan blue dye using a syringe-based differential hydrostatic pressure. Bright field microscope images show the interior of the microchamber during the fluid flow (S3 Fig).

### 3D Surface Topography and Nano-roughness of the Niche

**Fig 3:**
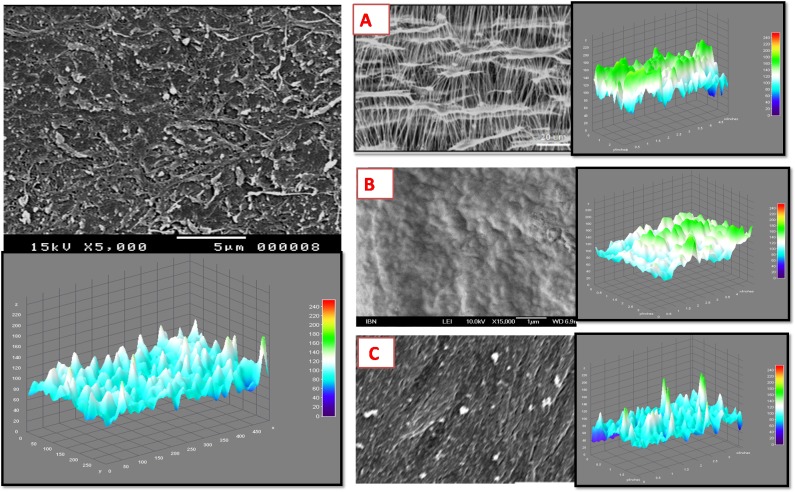
3D Nano-topography of the Decellularized hAM Based Platform. The left panel of this Fig shows a SEM image of the hAM at x5000 magnification. It demonstrates the nano-scale topography of the membrane after decellularization. Below the original SEM image, a 3D- surface plot is obtained by ImageJ which illustrates the peaks and valleys distributed homogenously throughout the surface. On the right panel, 3 examples of synthetic scaffolds are presented along with their corresponding 3D surface plotting using the same ImageJ surface plotting parameters. (A) demonstrates a commercial 3D culture plate coating substrate composed of Polytetrafluoroethylene (PTFE) polymer modified with an amphiphilic polymer and collagen. (B) shows a SEM image of a 3D commercial scaffold (at x15000 magnification) composed of inert hydroxypropyl cellulose to form a sponge like structure. (C) demonstrates a synthetic scaffold fabricated from polypyrrole (PPy) and poly(styrene sulfonate) (PSS) interwoven within a PCL matrix[43]

**Fig 4:**
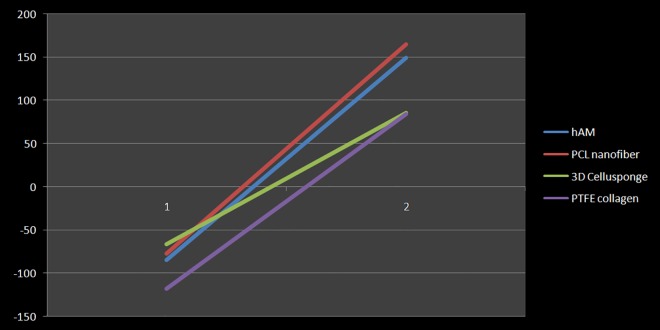
Surface Characterization of the Decellularized hAM Based Platform. A line diagram illustration of the 3D surface roughness parameter (R) of each scaffold characterized using *SurfaceJ*. It shows the span between the lowest valley (1), and the highest peak (2) in each scaffold. Detailed SurfaceJ characterization is shown in supplementary information (S1 File).

The SEM imaging demonstrated the topography of the decellularized membrane which composed the substrate of the described prototypes. No apparent damage to the basement membrane was observed at various magnifications. At higher magnification (x5000), the 3D ultra-topography of the decellularized membrane was well evident at the nano-scale. The 3D surface plotting using ImageJ enabled the representation of the 3D architectural arrangement of the membrane. The decellularized hAM showed a more homogenous distribution of the 3D patterns compared to other 3D synthetic scaffolds (Fig 3). Furthermore, using the specialized SurfaceJ plugin, we were able to quantitatively map the surface topography of the hAM and to confirm that its nano-roughness characteristics are comparable to those of other 3D synthetic scaffolds sampled in this study (Fig 4). The surface characteristics of hAM and other biomaterials, including multi-parameter quantitative analysis of height and depth distribution of surface projections, are detailed in the supplementary information (S1 File).

### Proliferation and Differentiation Studies

**Fig 5:**
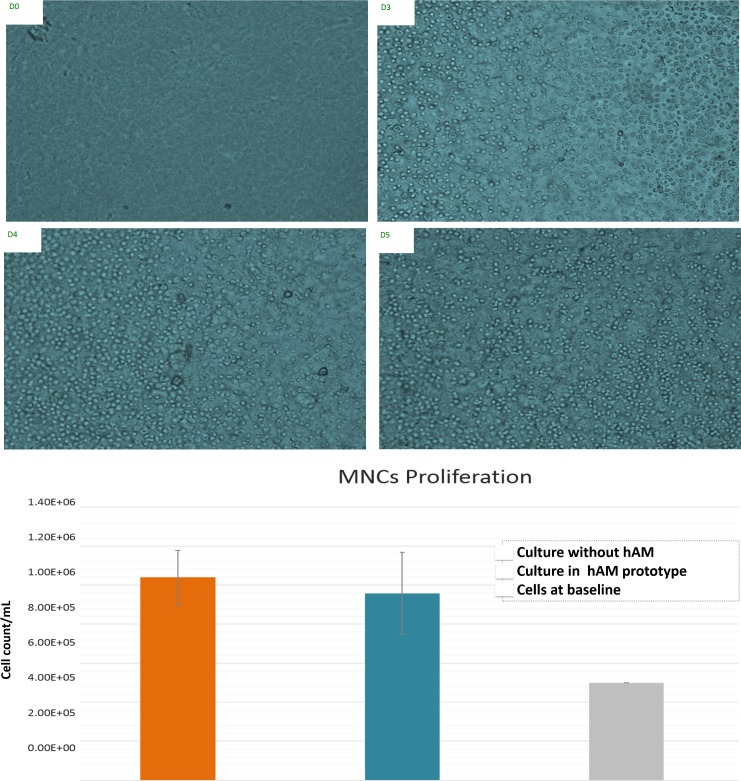
Human Umbilical Cord Blood Mononuclear Cell (MNCs) Culture. This figure shows progressive proliferation of (MNCs) obtained from the umbilical cord blood and cultured on a 6-well plate lined with hAM (Prototype 1). D0 stands first day immediately after culture. D3, 4, 5 represent cell proliferation on days 3, 4 and 5 respectively. The lower panel shows a bar chart for cell proliferation of the MNCs, in another run of experiments, after 7 days of culture in the hAM coated and non-coated plates. The grey bar represents the original number of cells per ml before culture. (Cell images were obtained by an inverted microscope (x40). (*Cropping and coloring of image was slightly done for aesthetic purposes without affecting presented data*)

The proliferation of umbilical cord blood MNCs cultured in the amniotic membrane platform (prototype 1); was observed in repeat experiments. MNCs proliferation was evaluated on the 4^th^ and 7^th^ days in both the hAM coated and non-coated plates. MNCs survived and exhibited healthy proliferation under both experimental conditions, and the number of cells on the 7^th^ day of culture, was not significantly different in the compared groups (The average cell count in the hAM prototype and the control group was 9.57E+05 cells/ml and 1.04E+06 cells/ml, with a CV% of 22% and 13%, respectively; *p* = 0.25) (Fig 5).

When another run of MNCs cell culture was left under non-sterile conditions, we observed bacterial colonies that developed on day 4 in the regular non-coated wells. We intentionlly maintained the 2 groups for observation under the same conditions for further 7 days, and serial images were obtained till day 11. The number of colonies increased progressively over the following days in the regular, non-treated plates, while cells in the hAM coated plates remained distinctively sterile (S4 Fig).

**Fig 6:**
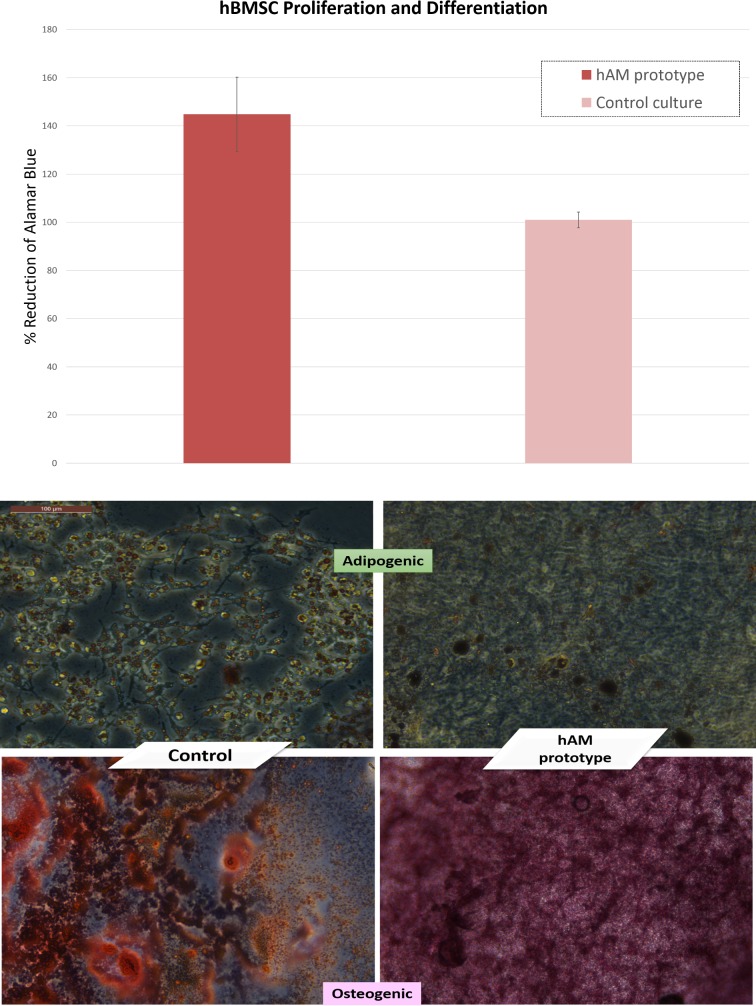
Proliferation and Differentiation of Human Bone Marrow Stem Cells (hBMSC) in the Biomimetic Platform. The top panel shows a graph depicting proliferation of the hBMSCs as expressed by the relative % reduction of the Alamar Blue reagent using the absorbance values in the study group compared to the control group. The lower panel presents inverted microscope images (x10) of adipogenic (top) and osteogenic differentiation (bottom) of hBMSC grown on prototype 1(hAM coated plates) and control culture plate.

For hBMSC culture, the Alamar Blue assay showed significant increase in proliferation of the hBMSC cultured in the hAM based prototype compared to the control group of wells (Relative cell proliferation based on % reduction of the dye based on the absorbance values was at an average of 144% ± CV% of 15% in the hAM based culture compared to the control culture (100% ± 3%), *p* = 0.009). hBMC showed effective differentiation into both adipogenic and osteogenic lineages upon induction by the corresponding differentiation media as shown in (Fig 6)

### Breast Cancer Explant Culture

Progressive proliferation of the breast cancer tissue explants was observed in the regular well plate. Proliferation started after the first week and continued progressively for up to 7 weeks of observation. In contrast, no cell proliferation was observed in the hAM-based platform during the same observation period (S5 Fig).

### Cost Effectiveness of the Biomimetic Platform

When compared with a sample of commercial 3D culture platforms available in the international market, there was a considerable economic advantage of our proposed models (Table 1). While the average cost of the commercially available 3D coated plates is $65, our prototype 1 platform only costs less than one dollar to manufacture, given that the hAM is collected for free as a regular delivery by product. Our platform thus costs at least 40–50 times lower than commercial 3D culture plates. The more complex prototypes, prototypes 2 and 3, can be manufactured for a slightly higher cost of around $5, which includes costs involved in prototype design, fabrication and operation of the dynamic flow. It is to be noted that the proposed hAM based models provide indefinite number of matrix proteins compared to only one or two proteins typically included in the equivalent commercial culture plates. Moreover, most of the synthetic commercial platforms require technically demanding fabrication methods such as nanofabrication techniques, which add significantly to the cost, whereas, the simple composition of the presented prototypes enable basic and less funded research laboratories to reproduce or adapt the method to their different needs at the lowest cost.

**Table 1:** A Comparison of Cost of Example Commercial 3D Culture Platforms.

Platform	Price*	Manufacturer	Main composition
Alvetex™ 3D Cell Growth Plates	$211.89-$759.92/packs of 2–10 plates.	Thermo Scientific	Synthetic polystyrene scaffold.
Cellusponge-Collagen	$ 2000 (pack of 24 plates)	InvitroCue	Plates coated with inert hydroxypropyl cellulose covered with collagen.
Z764086 SIGMA MicroTissues® 3DPetri Dish	$500 (pack of 6 plates)	MicroTissues Inc.	Scaffold free, agraose molded in honey-comb shaped wells.
PureCoat™	$ 319 (pack of 10)	Corning	A cell culture flask coated with fibronectin peptide.
Nanofiber cell culture dish	$ 29 /each plate	Sigma	Nanofiber cell culture dish, with aligned nanofibers.
Proposed study models	$1$/ plate (authors estimation)**	NA	As described in the manuscript

*Prices were obtained either from manufacturer website or main vendors in the biotechnology field.

** This price is estimated for the static 3D culture model (prototype 1) to be comparable with the commercial models.

## Discussion

Herein, we present a model for a biomimetic niche, for *in vitro* cell culture applications, which is well suited for stem cell culture. In this model, we included a number of key niche factors representative of the native cell microenvironment.

We selected the hAM to provide the biological element of the presented cell niche. The hAM has a potent niche effect based on robust clinical and research evidence. It has been used as a surgical graft for decades. Although the use of hAM, for the most part, has focused on ocular applications[24, 25], other uses emerged to expand its applicability as a graft for treating chronic wounds [26] or as a substrate for culturing neural cells[27]. These applications rely on the widely acknowledged properties of the hAM in enhancing proliferation of cells of ectodermal origin. Lately, the hAM was applied in vascular surgery showing potential in supporting proliferation of cells of endodermal origin, such as endothelial [28] or urothelial cells [29]. In the latter, it was noted that the loose connective tissue interface of the membrane could promote faster proliferation compared to the classically used basement membrane side. In addition, the hAM showed pro-apoptotic and anti-angiogenic features that could support its anti-inflammatory effect[30]. Such diverse immunological and growth enhancing attributes of the hAM is well-matched for a multi-purpose culture system. For instance, the anti-angiogenic effect fits well with limbal stem cell culture applications where angiogenesis is not a desirable outcome, unlike the case of wound healing where angiogenesis is a crucial repair mechanism.

Numerous methods were used for the decellularization of the hAM before its use as a grafting biomaterial, including the use of urea, EDTA, thermolysin, sodium dodecyl sulfate (SDS), or only mechanical scraping. These methods required soaking of the membrane in the treating reagents which may affect the integrity of the stroma and cause damage of its matrix proteins, or may render the membrane fragile and difficult to handle. The brief alkaline treatment method we followed proved to be simple, efficient and reproducible [19]. Using our designed cell crown enabled the reproducible preparation of hAM readily in short time.

In this study, we used human umbilical cord blood MNCs, a rich source of multipotent stem cells [31, 32], as well as hBMSCs. There was no significant difference in proliferation of the MNCs when cultured on hAM compared to traditional culture plates. On the other hand, there was a significant increase in proliferation of the hBMSC in the hAM coated plates compared to the control plates. These findings provide basic evidence of the safety of the hAM based culture system for diverse stem cell applications. Few reports in the literature have addressed the effect of hAM on cells of mesodermal origin, including the MNCs or the hBMSCs. One study showed an anti-proliferative effect of a decellularized hAM on the peripheral adult blood MNCs[33]. In reports of hAM transplantation into defective cornea, it was proposed that the epithelial limbal stem cells may migrate to the basement membrane while the mesenchymal stem cells migrate inside the membrane matrix[34]. We excluded the trapping of cells underneath the membrane surface by performing thorough wash of the membrane before cell counting. The membrane was then re-examined and no attached cells were observed.

For the purpose of demonstrating flexibility of this platform, we have used a decellularized hAM based platform for the culture of MNCs and hBMSC, while we used an intact hAM platform for the explant breast cancer tissue culture. Although the majority of epithelial culture studies report the use of a decellularzed membrane, however, we believe that the selection of an intact, decellularized or partially decellularized membrane can be based on the type of cells, and should be tailored to the study purpose. For example, the presence of co-cells in the membrane may theoretically enhance desired cell to cell interactions. It was found earlier that epithelium enhancing growth factors such as the epidermal growth factor (EGF) and b-FGF were higher in the intact hAM compared to the decellularized one, as they are primarily produced by the epithelial cells [14]. This may explain its proliferative enhancing effect on the benign corneal epithelium. Nonetheless, other studies showed that the amnion may produce cytotoxic cytokines and inhibitory factors for epithelial cancer cells. Although our findings of the lack of proliferation in the breast cancer explant culture may indicate similar anti-cancer effect, recently reported in several studies [35–39], this finding requires follow up experimentation dedicated to the effect of hAM culture with different types of cancers and larger number of patients’ samples.

In the present study, we used fresh non-sterilized and minimally treated amniotic membrane. There is some evidence that preservatives, aggressive decellularization procedures and radiation may affect the functional properties of the membrane. For example, the anti-angiogenic and anti-endothelial effects were found to be significantly higher in non-sterilized amniotic membranes compared to irradiated ones[40]. In addition, we observed that cryogenic preservation of the hAM made it considerably difficult to handle and more liable to tears compared to the fresh hAM.

Based on an incidental observation of a few contaminating colonies in a regular culture plate, but not in the hAM coated plates, we performed a prospective study to compare both groups for 11 days. A progressive increase in the contaminating colonies continued in the control group, while none has occurred in any of the hAM coated plates (Fig 6). It is to be re-emphasized that the hAM was not sterilized in this study (neither by gamma irradiation or sterilizing solutions), while the regular non-coated plates were sterile (gamma radiation or electron beam are usually used by the manufacturer). Nevertheless, the hAM coated plates showed a distinct ability to maintain an infection-free environment for the proliferating cells throughout the observational study. This outcome is in agreement with the known antibacterial effect of the hAM either *in vivo* or *in vitro*[41, 42]. These data were based on the ability of the membrane to suppress bacterial growth in bacterial culture media or in animal models. However, we found no similar investigations for human cell culture.

The second niche factor proposed in our model is the 3D topography. The decellularized hAM has maintained a homogenous 3D nanoscale roughness that is quantitatively and qualitatively comparable to sophisticated 3D synthetic commercial substrates. In the PDMS prototype 2, the 3D features of the membrane could be further enhanced using the moldability of PDMS which allows printing composite 3D patterns. PDMS is a popular organic elastomer in microfabrication technologies. It has the advantage of being inert, non-toxic, and transparent and it allows the exchange of gases, which is an advantage for cultured cells. It allows the flexible molding of unlimited number of designs and can be prepared with minimal technical expertise.

The fluid dynamic factor is the third feature of our model. We have established methods for introducing a continuous fluid flow in the macroscale and microscale versions of the model to mimic the dynamicity of the extracellular fluid. As microfluidics is gaining increased access to cell culture applications, the incorporation of the hAM into a microfluidic chamber provides the joint benefits of the distinct biological composition of the hAM together with the ability to precisely control a gradient of culture media and flow rates across the niche. This is the first report of such microfluidics design. We are currently working on optimizing the cell culture conditions in these models. We also suggest that adding xeno-free media such as the amniotic fluid or platelet rich plasma can be ideal for enhancing this system’s biomimicry profile.

## Conclusion

Herein, we present a method for preparing a biomimetic cell culture niche. Our platform provides an integrated model that combines native biological, topographic and mechanical factors in one simple low cost setup. This model can be applied into unrestricted number of platform designs to suit diverse cell culture applications.

## Supporting Information

S1 Fig Decellularization Method of the hAM. A homemade cell crown is designed to allow homogenous distribution of reagents and easy handling of the amniotic membrane (top and side views). Top right, manual separation was done at line of cleavage between the amnion and the chorion.
(TIF)

S2 Fig Partial hAM Decellularization. The amniotic membrane after treatment with trypsin followed by NaOH. The image shows partial decellularization of the membrane. Methylene blue stained cells are seen on the right side of the image.
(TIF)

S3 Fig Microfluidics-hAM based platform–Prototype 3. Inner view of the hAM integrated microfluidic chamber. A trypan blue flow was introduced through the inlet microchannels to demonstrate the decellularized membrane inside the micro-chamber.
(TIF)

S4 Fig Observed Anti-infective Property of the hAM Based MNCs Culture. This figure shows umbilical cord MNCs cultured in the hAM coated (bottom panel) and similar non-coated regular plates (top panel). Cell culture observations on days 0, 4, 6 and 11 were compared between both groups. Contaminating bacterial colonies started on day 4 on the non-coated plates, while none occurred in the hAM group throughout the observation study. (*Cropping and coloring of image was slightly done for aesthetic purposes without affecting presented data*)
(TIF)

S5 Fig Human Breast Cancer Explant Culture in an Intact hAM Based Platform. Breast cancer explant culture is shown in prototype 1, lined by an intact hAM. The upper panel shows the breast explant tissue on the hAM on culture days 0, 7 and 30. The lower panel shows the successive proliferation of epithelial cells originating from the explants cancer tissue in a regular plate. (Cell images were obtained by an inverted microscope (x20 and x40). (*Cropping and coloring of image was slightly done for aesthetic purposes without affecting presented data*)
(TIF)

S1 File SurfaceJ Topography Characterization. This data file is produced by SurfaceJ to show a quantitative analysis of the surface topography of the decellularized hAM in comparison with other synthetic scaffolds. It shows also the processing parameters used for the analysis.
(XLSM)

S2 File Absorbance Equation. This equation was used to calculate the relative cell proliferation based on absorbance measurements of the Alamar blue reagent (Resazurin) at 570 nm, using 600 nm as a reference wavelength.
(PDF)

## References

[1] Ravi M, Paramesh V, Kaviya SR, Anuradha E, Solomon FD. 3D cell culture systems: advantages and applications. Journal of cellular physiology. 2015;230(1):16–26. Epub 2014/06/10. 10.1002/jcp.24683 24912145

[2] Walasek MA, van Os R, de Haan G. Hematopoietic stem cell expansion: challenges and opportunities. Annals of the New York Academy of Sciences. 2012;1266:138–50. Epub 2012/08/21. 10.1111/j.1749-6632.2012.06549.x 22901265

[3] Eggenhofer E, Benseler V, Kroemer A, Popp FC, Geissler EK, Schlitt HJ, et al Mesenchymal stem cells are short-lived and do not migrate beyond the lungs after intravenous infusion. Frontiers in immunology. 2012;3:297 Epub 2012/10/12. PubMed Central PMCID: PMC3458305. 10.3389/fimmu.2012.00297 23056000 PMC3458305

[4] Scadden David T. Nice Neighborhood: Emerging Concepts of the Stem Cell Niche. Cell. 2014;157(1):41–50. 10.1016/j.cell.2014.02.013 24679525 PMC4161226

[5] Plaks V, Kong N, Werb Z. The Cancer Stem Cell Niche: How Essential Is the Niche in Regulating Stemness of Tumor Cells? Cell Stem Cell. 2015;16(3):225–38. 10.1016/j.stem.2015.02.015 25748930 PMC4355577

[6] Guilak F, Cohen DM, Estes BT, Gimble JM, Liedtke W, Chen CS. Control of stem cell fate by physical interactions with the extracellular matrix. Cell Stem Cell. 2009;5(1):17–26. Epub 2009/07/03. PubMed Central PMCID: PMC2768283. 10.1016/j.stem.2009.06.016 19570510 PMC2768283

[7] Eberwein P, Reinhard T. Concise Reviews: The Role of Biomechanics in the Limbal Stem Cell Niche: New Insights for Our Understanding of This Structure. STEM CELLS. 2015;33(3):916–24. 10.1002/stem.1886 25410061

[8] Cooper LJ, Kinoshita S, German M, Koizumi N, Nakamura T, Fullwood NJ. An investigation into the composition of amniotic membrane used for ocular surface reconstruction. Cornea. 2005;24(6):722–9. 16015093 10.1097/01.ico.0000154237.49112.29

[9] Lutolf MP, Gilbert PM, Blau HM. Designing materials to direct stem-cell fate. Nature. 2009;462(7272):433–41. 10.1038/nature08602 19940913 PMC2908011

[10] Westekemper H, Figueiredo FC, Siah WF, Wagner N, Steuhl KP, Meller D. Clinical outcomes of amniotic membrane transplantation in the management of acute ocular chemical injury. The British journal of ophthalmology. 2016. Epub 2016/05/07. 10.1136/bjophthalmol-2015-308037 27150827

[11] Wells WJ. Amniotic Membrane for Corneal Grafting. British medical journal. 1946;2(4477):624.

[12] Rocha SCM, Baptista CJM. Biochemical Properties of Amniotic Membrane. Amniotic Membrane: Springer; 2015 p. 19–40.

[13] Deolinda de Oliveira Pena J, Melo GB, Gomes JA, Haapalainen EF, Komagome CM, Santos NC, et al [Ultrastructural and growth factor analysis of amniotic membrane preserved by different methods for ocular surgery]. Arquivos brasileiros de oftalmologia. 2007;70(5):756–62. Epub 2007/12/25. 18157297 10.1590/s0004-27492007000500006

[14] Koizumi N, Inatomi T, Sotozono C, Fullwood NJ, Quantock AJ, Kinoshita S. Growth factor mRNA and protein in preserved human amniotic membrane. Current Eye Research. 2000;20(3):173–7. 10694891

[15] Stock SJ, Kelly RW, Riley SC, Calder AA. Natural antimicrobial production by the amnion. American Journal of Obstetrics and Gynecology. 2007;196(3):255.e1–.e6. 10.1016/j.ajog.2006.10.908 17346544

[16] Solomon A, Rosenblatt M, Monroy D, Ji Z, Pflugfelder SC, Tseng SC. Suppression of interleukin 1α and interleukin 1β in human limbal epithelial cells cultured on the amniotic membrane stromal matrix. British Journal of Ophthalmology. 2001;85(4):444–9. 10.1136/bjo.85.4.444 11264135 PMC1723909

[17] Ueta M, KWEON MN, Sano Y, Sotozono C, Yamada J, Koizumi N, et al Immunosuppressive properties of human amniotic membrane for mixed lymphocyte reaction. Clinical & Experimental Immunology. 2002;129(3):464–70. 12197887 10.1046/j.1365-2249.2002.01945.x PMC1906465

[18] Li H, Niederkorn JY, Neelam S, Mayhew E, Word RA, McCulley JP, et al Immunosuppressive factors secreted by human amniotic epithelial cells. Investigative ophthalmology & visual science. 2005;46(3):900–7. 15728546 10.1167/iovs.04-0495

[19] Saghizadeh M, Winkler MA, Kramerov AA, Hemmati DM, Ghiam CA, Dimitrijevich SD, et al A simple alkaline method for decellularizing human amniotic membrane for cell culture. PloS one. 2013;8(11):e79632 Epub 2013/11/16. PubMed Central PMCID: PMC3827346. 10.1371/journal.pone.0079632 24236148 PMC3827346

[20] Yam GH-F, Zhang T, Riau AK, Beuerman R, Tan D, Mehta J. The Effect of Amniotic Membrane De-epithelialization Method on its Biological Properties and Ability to Promote Limbal Epithelial Cell Culture. Investigative ophthalmology & visual science. 2013;54(15):553-. 10.1167/iovs.12-10805 23580491

[21] Ahmed O, Abdellah H, Elsayed M, Abdelgawad M, Mousa NA, El-Badri N, editors. Tissue dissociation miniaturized platform for uterine stem cell isolation and culture. Biomedical Engineering Conference (CIBEC), 2014 Cairo International; 2014 11–13 Dec. 2014.

[22] Pham PV, Vu NB, Pham VM, Truong NH, Pham TL, Dang LT, et al Good manufacturing practice-compliant isolation and culture of human umbilical cord blood-derived mesenchymal stem cells. Journal of translational medicine. 2014;12:56 Epub 2014/02/26. PubMed Central PMCID: PMC3939935. 10.1186/1479-5876-12-56 24565047 PMC3939935

[23] Pei XF, Noble MS, Davoli MA, Rosfjord E, Tilli MT, Furth PA, et al Explant-cell culture of primary mammary tumors from MMTV-c-Myc transgenic mice. In vitro cellular & developmental biology Animal. 2004;40(1–2):14–21. Epub 2004/06/08. 15180438 10.1290/1543-706X(2004)40<14:ECOPMT>2.0.CO;2

[24] Kim JC, Tseng SC. Transplantation of preserved human amniotic membrane for surface reconstruction in severely damaged rabbit corneas. Cornea. 1995;14(5):473–84. 8536460

[25] Meller D, Pires R, Tseng S. Ex vivo preservation and expansion of human limbal epithelial stem cells on amniotic membrane cultures. British Journal of Ophthalmology. 2002;86(4):463–71. 11914219 10.1136/bjo.86.4.463 PMC1771095

[26] Mrugala A, Sui A, Plummer M, Altman I, Papineau E, Frandsen D, et al Amniotic membrane is a potential regenerative option for chronic non-healing wounds: a report of five cases receiving dehydrated human amnion/chorion membrane allograft. International Wound Journal. 2015:n/a-n/a. 10.1111/iwj.12458 PMC7950060 25974156

[27] Davis G, Blaker S, Engvall E, Varon S, Manthorpe M, Gage F. Human amnion membrane serves as a substratum for growing axons in vitro and in vivo. Science. 1987;236(4805):1106–9. 3576223 10.1126/science.3576223

[28] Peirovi H, Rezvani N, Hajinasrollah M, Mohammadi SS, Niknejad H. Implantation of amniotic membrane as a vascular substitute in the external jugular vein of juvenile sheep. Journal of Vascular Surgery. 2012;56(4):1098–104. 10.1016/j.jvs.2012.02.036 22560305

[29] Jerman UD, Veranič P, Kreft ME. Amniotic membrane scaffolds enable the development of tissue-engineered urothelium with molecular and ultrastructural properties comparable to that of native urothelium. Tissue Engineering Part C: Methods. 2013;20(4):317–27. 23947657 10.1089/ten.tec.2013.0298 PMC3968885

[30] Mamede AC, Carvalho MJ, Abrantes AM, Laranjo M, Maia CJ, Botelho MF. Amniotic membrane: from structure and functions to clinical applications. Cell and tissue research. 2012;349(2):447–58. Epub 2012/05/18. 10.1007/s00441-012-1424-6 22592624

[31] Lee OK, Kuo TK, Chen W-M, Lee K-D, Hsieh S-L, Chen T-H. Isolation of multipotent mesenchymal stem cells from umbilical cord blood. Blood. 2004;103(5):1669–75. 10.1182/blood-2003-05-1670 14576065

[32] Jin HJ, Bae YK, Kim M, Kwon S-J, Jeon HB, Choi SJ, et al Comparative analysis of human mesenchymal stem cells from bone marrow, adipose tissue, and umbilical cord blood as sources of cell therapy. International journal of molecular sciences. 2013;14(9):17986–8001. 10.3390/ijms140917986 24005862 PMC3794764

[33] Garfias Y, Zaga-Clavellina V, Vadillo-Ortega F, Osorio M, Jimenez-Martinez MC. Amniotic membrane is an immunosuppressor of peripheral blood mononuclear cells. Immunological investigations. 2011;40(2):183–96. Epub 2010/11/18. 10.3109/08820139.2010.532266 21080833

[34] Tseng SC, Espana EM, Kawakita T, Di Pascuale MA, Li W, He H, et al How does amniotic membrane work? The ocular surface. 2004;2(3):177–87. Epub 2007/01/12. 17216089 10.1016/s1542-0124(12)70059-9

[35] Kang NH, Hwang KA, Kim SU, Kim YB, Hyun SH, Jeung EB, et al Potential antitumor therapeutic strategies of human amniotic membrane and amniotic fluid-derived stem cells. Cancer gene therapy. 2012;19(8):517–22. Epub 2012/06/02. 10.1038/cgt.2012.30 22653384

[36] Mamede A, Laranjo M, Carvalho MJ, Abrantes AM, Pires AS, Brito AF, et al Effect of Amniotic Membrane Proteins in Human Cancer Cell Lines: An Exploratory Study. J Membrane Biol. 2014;247(4):357–60. 24577414 10.1007/s00232-014-9642-3

[37] Kang N-H, Yi B-R, Lim SY, Hwang K-A, Baek YS, Kang K-S, et al Human amniotic membrane-derived epithelial stem cells display anticancer activity in BALB/c female nude mice bearing disseminated breast cancer xenografts. International journal of oncology. 2012;40(6):2022–8. 10.3892/ijo.2012.1372 22344679

[38] Nourazarian SM, Nourazarian A, Asl ER. Investigate the Inhibitory effect of Amniotic Membrane Proteins on HSP90 Gene Expression level in PC3 Prostate Cell line. Bull Env Pharmacol Life Sci. 2015;4:61–5.

[39] Mamede AC, Guerra S, Laranjo M, Carvalho MJ, Oliveira RC, Gonçalves AC, et al Selective cytotoxicity and cell death induced by human amniotic membrane in hepatocellular carcinoma. Med Oncol. 2015;32(12):1–11. 10.1007/s12032-015-0702-z 26507652

[40] SKOPIŃSKI P, Zdanowski R, Grzela T, KAMIŃSKI A, LEWICKI S, Skopińska-Różewska E, et al The influence of sterilized and non-sterilized amniotic dressings on the proliferation of endothelial cells in vitro. Central European Journal of Immunology. 2012;37(2):114.

[41] Kjaergaard N, Hein M, Hyttel L, Helmig R, Schønheyder H, Uldbjerg N, et al Antibacterial properties of human amnion and chorion in vitro. European Journal of Obstetrics & Gynecology and Reproductive Biology. 2001;94(2):224–9. 11165729 10.1016/s0301-2115(00)00345-6

[42] Robson MC, Krizek TJ. The effect of human amniotic membranes on the bacteria population of infected rat burns. Annals of surgery. 1973;177(2):144 4633388 10.1097/00000658-197302000-00003 PMC1355552

[43] Hardy JG, Cornelison RC, Sukhavasi RC, Saballos RJ, Vu P, Kaplan DL, et al Electroactive Tissue Scaffolds with Aligned Pores as Instructive Platforms for Biomimetic Tissue Engineering. Bioengineering. 2015;2(1):15–34. 28955011 10.3390/bioengineering2010015 PMC5597125

