# Supplementary figures and images for: A Cost-Effective Method to Assemble Biomimetic 3D Cell Culture Platforms
Source: PLoS One. 2016 Dec 9;11(12):e0167116. doi: 10.1371/journal.pone.0167116 (PMC5147837; doi:10.1371/journal.pone.0167116)

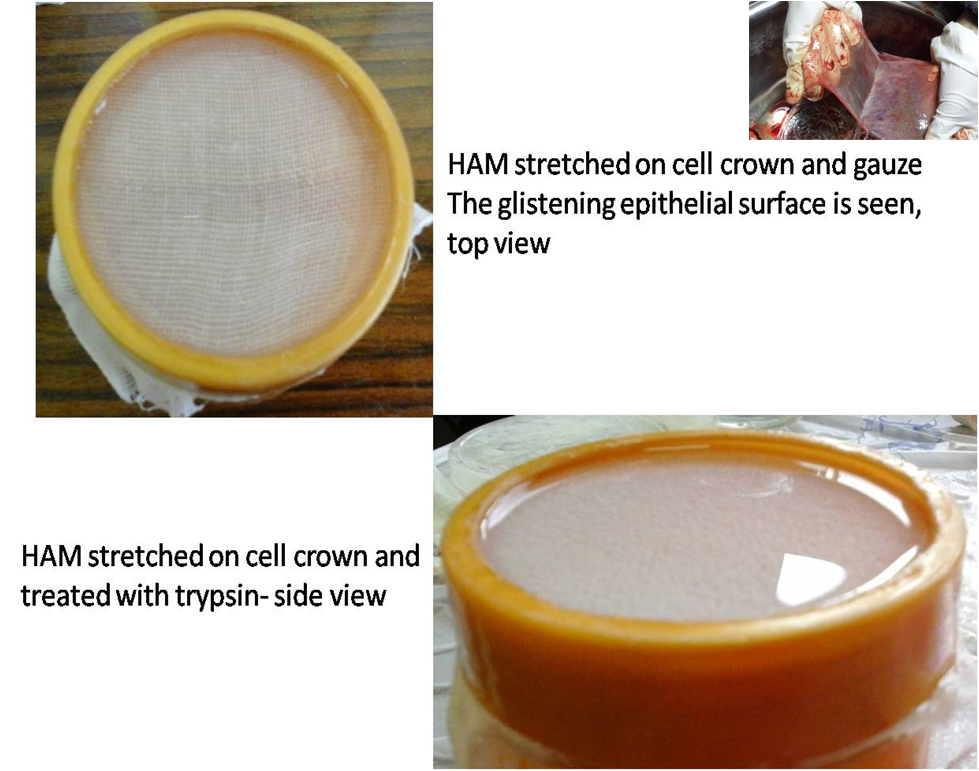

Supplement: S1 Fig — A homemade cell crown is designed to allow homogenous distribution of reagents and easy handling of the amniotic membrane (top and side views). Top right, manual separation was done at line of cleavage between the amnion and the chorion. (TIF) [file pone.0167116.s001.tif]

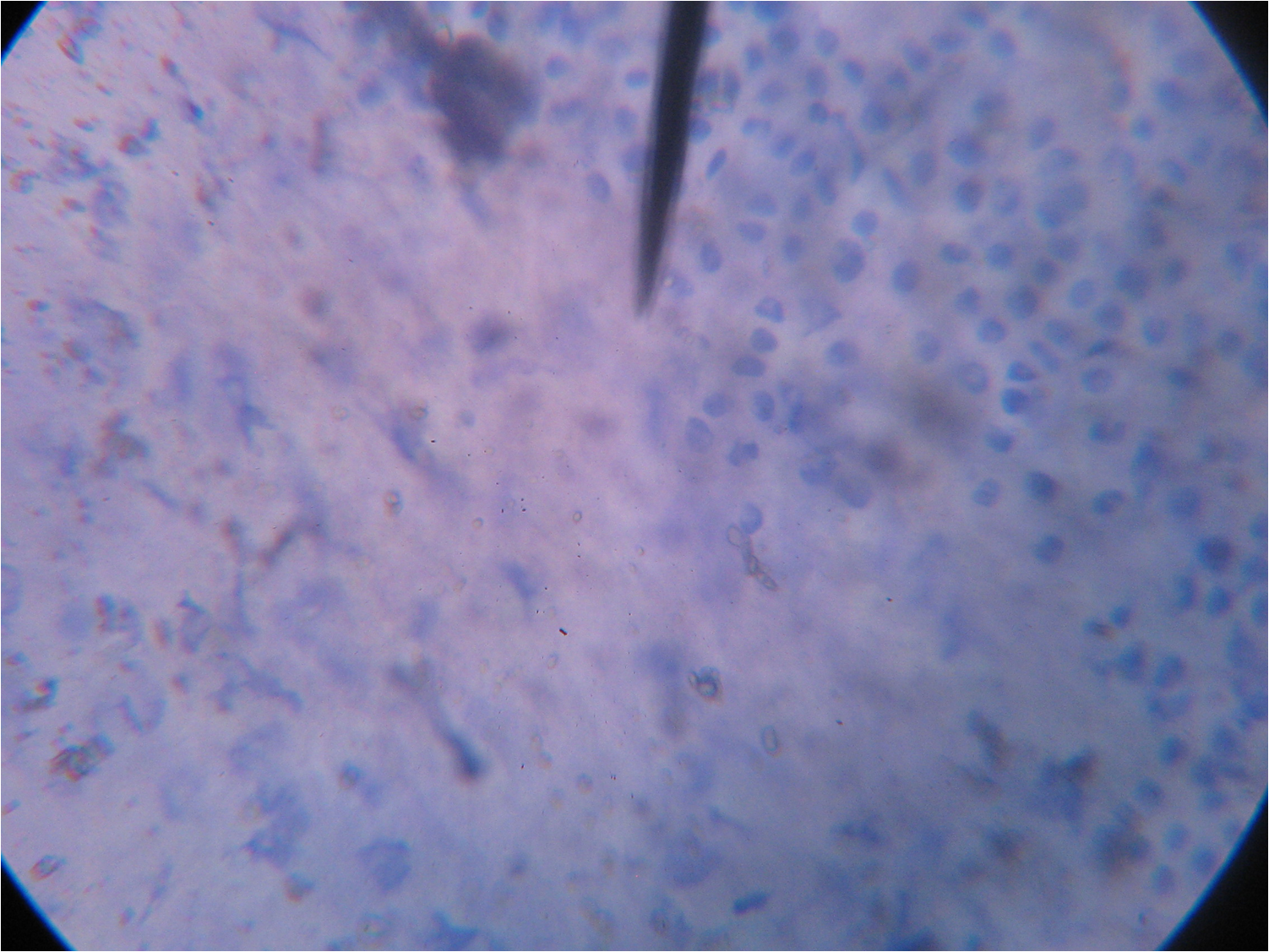

Supplement: S2 Fig — The amniotic membrane after treatment with trypsin followed by NaOH. The image shows partial decellularization of the membrane. Methylene blue stained cells are seen on the right side of the image. (TIF) [file pone.0167116.s002.tif]

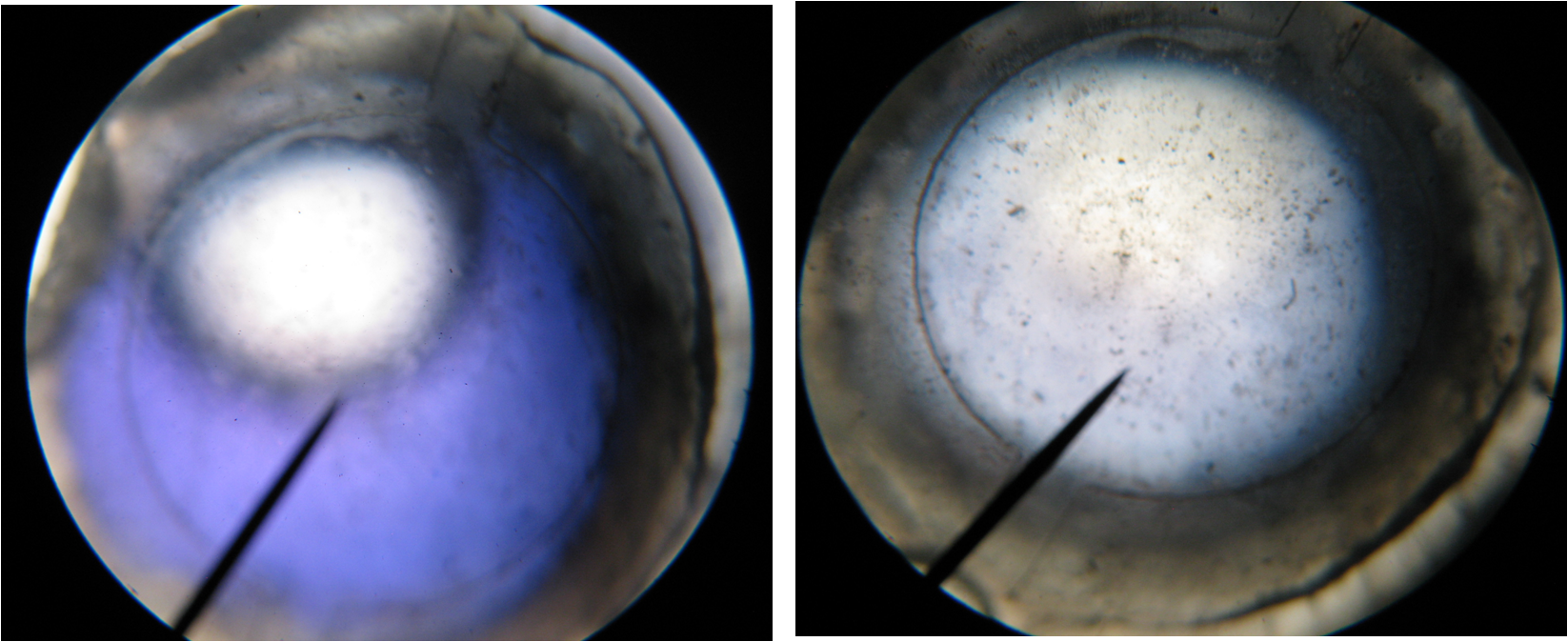

Supplement: S3 Fig — Inner view of the hAM integrated microfluidic chamber. A trypan blue flow was introduced through the inlet microchannels to demonstrate the decellularized membrane inside the micro-chamber. (TIF) [file pone.0167116.s003.tif]

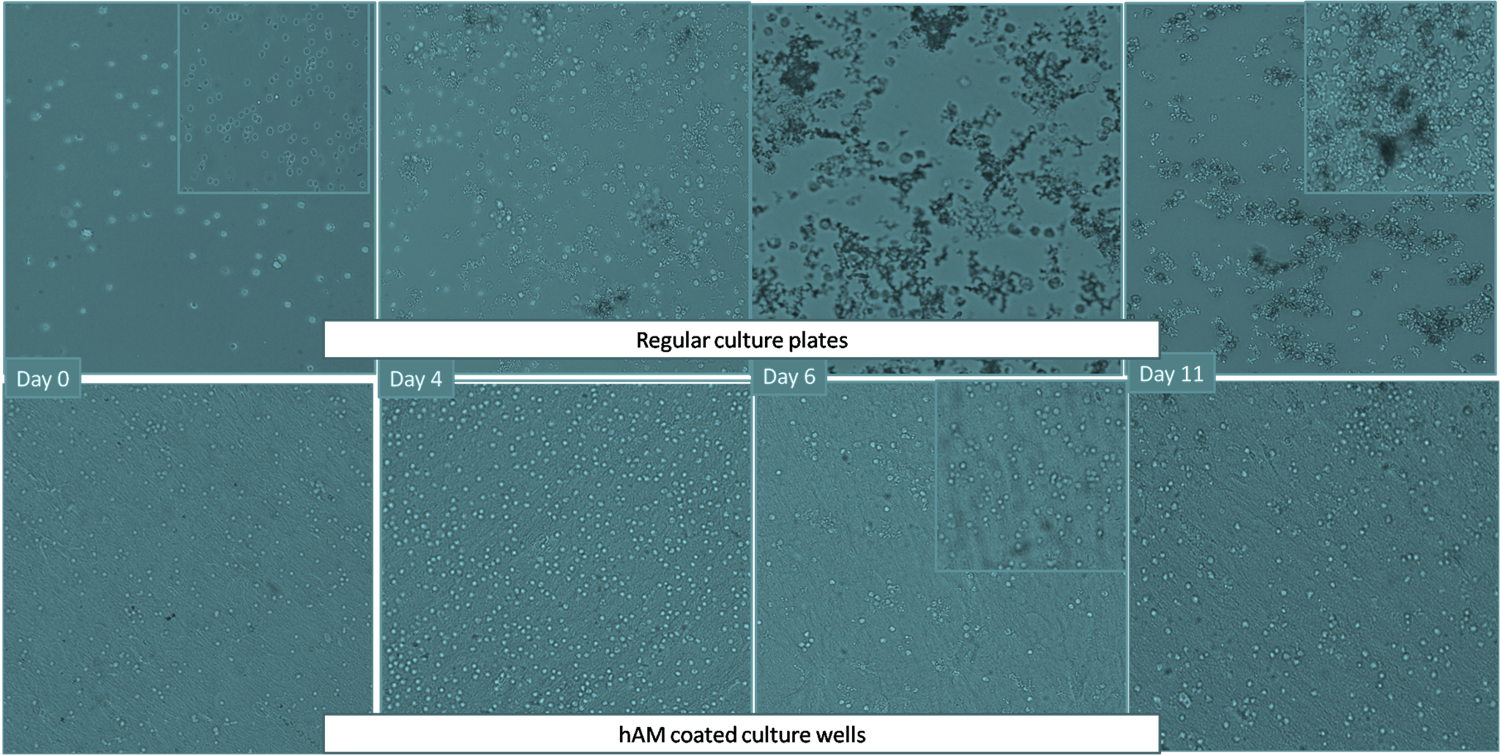

Supplement: S4 Fig — This figure shows umbilical cord MNCs cultured in the hAM coated (bottom panel) and similar non-coated regular plates (top panel). Cell culture observations on days 0, 4, 6 and 11 were compared between both groups. Contaminating bacterial colonies started on day 4 on the non-coated plates, while none occurred in the hAM group throughout the observation study. (Cropping and coloring of image was slightly done for aesthetic purposes without affecting presented data) (TIF) [file pone.0167116.s004.tif]

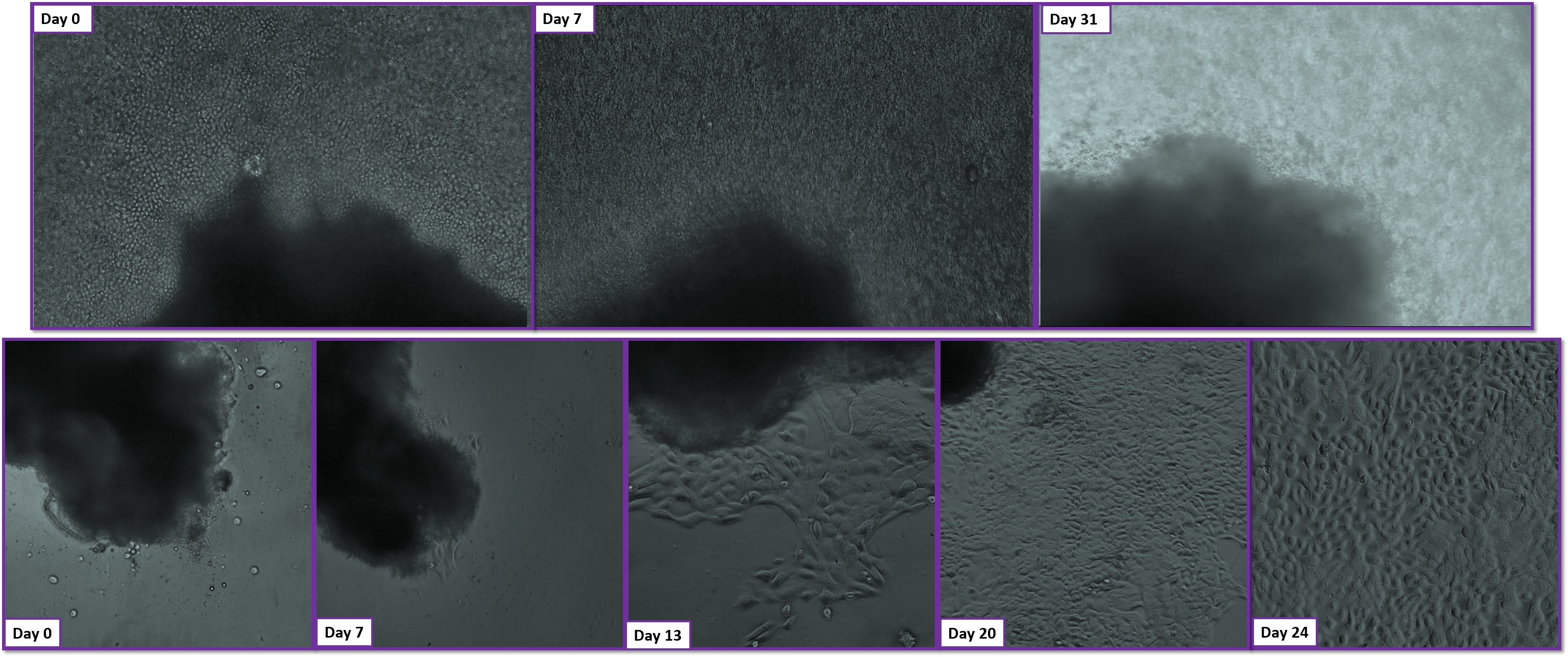

Supplement: S5 Fig — Breast cancer explant culture is shown in prototype 1, lined by an intact hAM. The upper panel shows the breast explant tissue on the hAM on culture days 0, 7 and 30. The lower panel shows the successive proliferation of epithelial cells originating from the explants cancer tissue in a regular plate. (Cell images were obtained by an inverted microscope (x20 and x40). (Cropping and coloring of image was slightly done for aesthetic purposes without affecting presented data) (TIF) [file pone.0167116.s005.tif]
