# Supplementary material for: A Cost-Effective Method to Assemble Biomimetic 3D Cell Culture Platforms
Source: PLoS One. 2016 Dec 9;11(12):e0167116. doi: 10.1371/journal.pone.0167116 (PMC5147837; doi:10.1371/journal.pone.0167116)
Supplement: S2 File — This equation was used to calculate the relative cell proliferation based on absorbance measurements of the Alamar blue reagent (Resazurin) at 570 nm, using 600 nm as a reference wavelength. (PDF) [file pone.0167116.s007.pdf]

Alamar Blue Assay

Calculating Proliferation Based on Absorbance

% Difference of study and control cells (% Reduction of OD between the study and control groups):

$$= (O_{600}X\ A_{570}) - (O_{570}XA_{600})/(O_{600}XP_{570}) - (O_{570}XP_{600})\ x\ 100$$

A570= OD of test sample (average OD values obtained in hAM groups) at 570 nm

A600= OD of test sample (average OD values obtained in hAM groups) at 600 nm

O570= Oxidation coefficient of Alamar Blue at 570 nm

O600= Oxidation coefficient of Alamar Blue at 600 nm

P570= OD of control cells (average OD values obtained in regular culture plates) at 570 nm

P600= OD of control cells (average OD values obtained in regular culture plates) at 600 nm
